# Beyond Hunger: The Structure, Signaling, and Systemic Roles of Ghrelin

**DOI:** 10.3390/ijms262210996

**Published:** 2025-11-13

**Authors:** Hlafira Polishchuk, Krzysztof Guzik, Tomasz Kantyka

**Affiliations:** 1 Department of Microbiology, Faculty of Biochemistry, Biophysics and Biotechnology, Jagiellonian University, Gronostajowa 7, 30-387 Krakow, Poland; polishchuk@uchicago.edu; 2 Department of Immunology, Faculty of Biochemistry, Biophysics and Biotechnology, Jagiellonian University, Gronostajowa 7, 30-387 Krakow, Poland; krzysztof.guzik@uj.edu.pl; 3 Malopolska Centre of Biotechnology, Jagiellonian University, Gronostajowa 7a, 30-387 Krakow, Poland

**Keywords:** ghrelin, GHSR1a, G protein-coupled receptor, GOAT

## Abstract

Our understanding of Ghrelin, an endogenous ligand of the growth hormone secretagogue receptor 1a (GHSR1a), has expanded from considering it to be a “hunger hormone” to a pleiotropic regulator of whole-body physiology. This review synthesizes the current advances spanning ghrelin biogenesis, signaling, and systems biology. Physiologically, preproghrelin processing and O-acylation by ghrelin O-acyltransferase (GOAT) generate acyl-ghrelin, a high-potency GHSR1a agonist; des-acyl ghrelin predominates in circulation and exerts context-dependent, GHSR1a-independent, or low-potency effects, while truncated “mini-ghrelins” can act as competitive antagonists. The emergence of synthetic ligands, agonists, antagonists, and reverse-agonists has provided the necessary tools to decipher GHSR1a activity. Recent cryo-EM structures of GHSR1a with peptide and small-molecule ligands reveal a bipartite binding pocket and provide a framework for biased signaling, constitutive activity, and receptor partner selectivity. Beyond the regulation of feeding and growth-hormone release, ghrelin modulates glucose homeostasis, gastric secretion and motility, cardiovascular tone, bone remodeling, renal hemodynamics, and innate immunity. Ghrelin broadly dampens pro-inflammatory responses and promotes reparative macrophage phenotypes. In the emerging scholarship on ghrelin’s activity in the central nervous system, ghrelin has been found to influence neuroprotection, stress reactivity, and sleep architecture, and has also been implicated in depression, Alzheimer’s disease, and substance-abuse disorders. Practical and transitional aspects are also highlighted in the literature: approaches for ghrelin stabilization; recent GHSR1a agonists/antagonists and inverse agonists findings; LEAP-2-based strategies; and emerging GOAT inhibitors. Together, structural insights and pathway selectivity position the ghrelin system as a druggable axis for the management of inflammatory diseases, neuropsychiatric and addiction conditions, and for obesity treatment in the post-GLP-1 receptor agonist era.

## 1. Introduction

In 1999, Kojima M. et al. utilized a screening method to associate orphan G protein-coupled receptors (GPCRs) with unknown ligands. Their method detected an increase in the intracellular calcium levels induced by agonists, leading to the purification of a 28-amino acid peptide from the gut. This peptide was identified as the natural ligand for the growth hormone secretagogue receptor 1a (GHSR1a) and was named “ghrelin”, derived from the Proto-Indo-European root “ghre”, signifying “grow” [1]. Since then, ghrelin has been discovered in many different organs, where it is involved in a wide range of processes and activities.

Comprehensive RT-PCR studies revealed that ghrelin mRNA is present in various tissues. The highest levels were observed in the stomach, whereas lower levels were detected in the liver, lung, kidney, and skeletal muscle [2]. In turn, at the protein level, ghrelin was detected in the stomach, small intestine, brain, cerebellum, pituitary, lungs, skeletal muscle, pancreas, salivary glands, adrenal gland, ovary, and testes, with concentrations ranging from 0.05 to 1.43 ng/mg of homogenate protein. The lungs and brain showed the highest levels. Protein was not found in the heart, liver, or kidneys, highlighting a potential discrepancy between gene and protein expression [2]. Recent studies have shown that ghrelin is also expressed in the oral cavity, since it was detected in saliva, gingival crevicular fluid (GCF), salivary glands, oral epithelial cells, and oral squamous cell carcinoma lesions. Its concentration is lowest in serum, while in GCF it is ~500-fold higher than in saliva [3,4].

The level of ghrelin-like immunoreactivity in plasma among healthy individuals, as determined by a specialized radioimmunoassay (RIA), stood at 117 ± 37 fmol/mL [5]. Ghrelin levels in the serum rise with age, irrespective of sex. They fluctuate considerably over the course of the day, peaking during sleep [6]. Unlike other gut hormones, ghrelin levels in the plasma rise during fasting and drop after eating [7]. Chronic high-calorie diets and obesity tend to lower plasma ghrelin levels in humans [7,8]. In rodents, long-term high-fat diets result in obesity and reduced stomach ghrelin production and secretion, simultaneously increasing the number of ghrelin-secreting cells [9]. However, the exact impact of increased adiposity on ghrelin production is unclear [10].

Ghrelin is reported to be the orexigenic peptide mainly secreted by X/A-like cells, a group of unique endocrine cells in the gastrointestinal tract [11]. Norepinephrine mediates the increase in ghrelin levels before a meal, whereas the decrease in ghrelin after a meal is regulated by glucose and insulin, with insulin contributing additively [12,13]. In addition to the reduced stomach production of ghrelin in obesity, decreased systemic levels have been observed in conditions such as male hypogonadism, untreated hyperthyroidism, polycystic ovary syndrome, and *H. pylori*-induced gastritis [14,15,16,17]. Conversely, elevated ghrelin levels are observed in conditions such as anorexia nervosa, in lean individuals, Prader–Willi syndrome, and following the eradication of *H. pylori* [17].

Two other research groups employed ghrelin-secreting cell lines from transgenic mice and primary cell cultures from mice and rats to demonstrate the mechanism underlying the regulation of ghrelin secretion [18,19]. The data demonstrate that a number of factors, including glucose, glucagon, dopamine, insulin, oxytocin, somatostatin, and long-chain fatty acids, directly act on ghrelin-producing cells, thereby controlling ghrelin release [12,18,19,20]. Recently, significant progress in our understanding of the ghrelin system was made, reflected by the determination of the ghrelin-bound GHSR1a structure, the functional description of the receptor’s biased signaling, and ongoing progress in structural and functional analysis of the ghrelin O-acyltransferase (GOAT). Together with our increased understanding of ghrelin’s role in the cardiovascular, reproductive, immune, and central nervous systems, and the emerging role of ghrelin in dopamine-related signaling and addiction, ghrelin is once again at the forefront of research. This review aims to provide a comprehensive, modern update on ghrelin biology.

## 2. Processing and Maturation of Ghrelin

**Figure 1:**
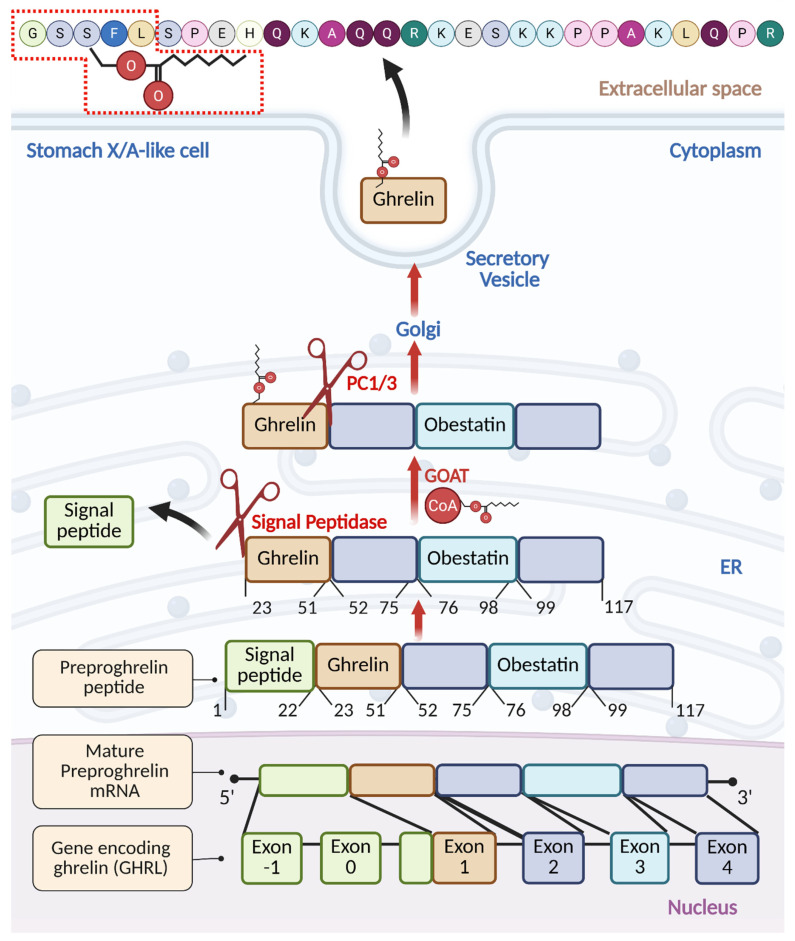
Intracellular processing of ghrelin. The human GHRL gene resides on the short arm of chromosome 3p25-26 and consists of six exons. The ghrelin gene undergoes transcription and splicing to generate mature mRNA. This mRNA is translated into pre-proghrelin, which then undergoes translation to yield a 117-amino acid peptide. Within the ER, this precursor undergoes a systematic cleavage process to produce the mature 28-amino acid form of ghrelin. Before secretion, unmature non-acylated ghrelin undergoes acylation by ghrelin-O-acyltransferase (GOAT) in the presence of CoA as an acyl donor, facilitating its binding to the growth hormone secretagogue receptor 1a (GHSR1a). The highlighted box indicates the minimal active n-terminal pentapeptide core of the ghrelin sequence necessary for GHSR1a activation. Created in BioRender. Kantyka, T. (2025) https://BioRender.com/0inw92w.

The ghrelin prepropeptide gene (*GHRL*) (Figure 1) consists of six exons and four introns, where mature preproghrelin mRNA is translated into preproghrelin, a 117-amino acid product [21,22]. One noteworthy feature of this construct is the presence of obestatin, a putative proteolytic fragment with activities opposite to those of ghrelin [22,23]. While ghrelin has an appetite-stimulating impact, obestatin, which is made up of 23 amino acids, is mainly linked to enhancing feelings of fullness [23]. Rats given obestatin had lower food intake, inhibited jejunal contractions, and gained less weight [24]. Human studies, however, could not demonstrate any connection between obestatin and anorexia or with weight loss or other dietary issues [25], indicating the potential species-dependent differences in obestatin recognition. We recommend a recent review of obestatin activities between different animal species [26].

To release mature ghrelin, the 117-amino acid precursor undergoes systematic proteolytic processing within the endoplasmic reticulum (ER) to form the 28-amino acid ghrelin. First, upon ER transport, signal peptidase removes the signal peptide at Arg^23^ [27]. Then, in the ER, ghrelin undergoes O-octanoylation at Ser^3^, catalyzed by ghrelin O-acyltransferase (GOAT), and is subsequently cleaved by the prohormone convertase PC1/3 at Arg^51^ to yield the active 28-amino acid acylated form of ghrelin (AG) [27,28]. The GOAT enzyme was initially identified to be expressed in the gut, and ghrelin *O*-octanoylation is essential for its binding to GHSR1a [28,29]. Recently, however, GOAT was detected in the urine and blood of prostate cancer (PC) patients, and, in these limited studies, its levels were reported to outperform PSA in predicting aggressive disease [30,31].

Some portion of preproghrelin undergoes C-terminal trimming by a carboxypeptidase-B-like enzyme, resulting in the 27-amino acid sequence of ghrelin lacking the C-terminal Arg^28^ [32]. This type of processing, commonly seen in other peptide hormones (e.g., endorphins [33], cholecystokinin [34]), often involves cleavage at or near the basic residues (Arg or Lys) [33,34]. The 27-residue isoform can also arise from alternative splicing. A splice variant, prepro-des-Gln14-ghrelin (116 residues), produces des-Gln14-ghrelin, a second endogenous GHSR ligand. However, des-Gln^14^-ghrelin appears to be less abundant in humans compared to rodents, where the ratio of ghrelin to des-Gln^14^-ghrelin precursors varies by species (e.g., ~5:1 in rats, ~6:5 in mice) [35].

Several studies have demonstrated the existence of truncated, bioactive ghrelin isoforms, collectively termed “mini-ghrelins”, that retain the essential N-terminal octanoyl modification and can modulate ghrelin receptor signaling [36,37,38]. Satou et al. identified activated protein C (APC), a serine protease found in bovine plasma, as a novel ghrelin endopeptidase. APC selectively cleaves human octanoylated ghrelin between Arg15 and Lys16, generating ghrelin_(1–15)_ as the predominant cleavage product. In vivo, the administration of ProTac, a snake-venom-derived pharmacological activator of APC, significantly enhanced this cleavage in mice [36].

Two additional shorter isoforms of mini-ghrelin have also been identified. Ghrelin_(1–14)_ likely arises via C-terminal trimming of ghrelin_(1–15)_, possibly mediated by plasma carboxypeptidases that remove terminal basic residues. The shortest characterized variant, ghrelin_(1–11)_, is generated by cleavage at the Arg11-Val12 bond in human ghrelin. This specific cleavage does not occur in rodents, where the corresponding residues are Lys-Ala, highlighting differences in protease recognition and substrate specificity [38].

Naturally occurring splice variants of the ghrelin gene (*GHRL*) have been identified across vertebrate species. A comparative genomic analysis of 77 species revealed that ghrelin exon 2 is symmetrical, allowing for it to be skipped without altering the downstream reading frame, resulting in a truncated preproghrelin transcript that encodes a 13-amino acid peptide followed by the obestatin-coding region. This exon 2-deleted isoform retains the overall structural organization of the preprohormone and has been detected in species such as mice and sheep, indicating evolutionary conservation and supporting its potential functional relevance also in the context of obestatin signaling [26,37].

Despite the preserved AG-like ability of mini-ghrelins to bind GHSR1a and inhibit calcium channel activity in vitro, mini-ghrelins do not elicit canonical ghrelin responses in vivo, such as food intake stimulation or hypothalamic c-Fos activation. Instead, these peptides act as competitive antagonists, inhibiting the binding and orexigenic effects of exogenous ghrelin [38]. This indeed confirms in vitro findings that identified the N-terminal region of the molecule as essential for receptor binding, but, at the same time, indicate more specific signaling pathways and hint at receptor-biased signaling.

The amino acid sequences of mammalian ghrelin exhibit significant conservation, especially in the N-termini, where the ten amino acids display a complete identity. The structural likeness and the ongoing necessity for acyl modification of the third residue indicate the pivotal importance of this N-terminal segment in the peptide’s function. Also, it is noteworthy that rat and human ghrelin differ by only two amino acid residues, and both show comparable potency in the activation of human GHSR1a [39,40]. The conservation of ghrelin-signaling systems in different organisms, even in the early branches of the vertebrate tree of life and with a high level of similarity, highlights the evolutionary conservation of ghrelin and indicates its fundamental role in energy regulation and the maintenance of homeostasis.

## 3. GOAT—A Single Enzyme for a Single Substrate

GOAT was independently discovered by two labs in 2008 [28,29]. It is a member of the membrane-bound-*O*-acyltransferase (MBOAT) family and is an integral membrane protein with 11 transmembrane α-helices. The GOAT structure remains uncharacterized due to inherent difficulties in the structural characterization of integral membrane proteins. Structures of other enzymes from the same family, DltB, a bacterial alanyltransferase solved by X-ray diffraction [41] and human sterol *O*-acyltransferase solved by cryo-EM [42], provide some insight into the enzyme mechanism and inhibitor design strategies for GOAT. In recent years, a topology-guided computational model of the GOAT structure was developed by James L. Hougland’s group [42]. GOAT forms an ellipsoidal cone embedded in the ER membrane, with a narrow end facing the lumen and the wide side directed toward the cytoplasm. Octanoyl-CoA is recruited from the cytoplasmic side and is not transported across the membrane, but rather the catalytic machinery of GOAT attaches the fatty acid chain to the lumen-delivered ghrelin within the channel.

It has been proposed that the unexpected biological activity of des-acyl ghrelin (DAG) may, at least in part, be mediated by re-acylation. Indeed, this emerging hypothesis was raised by several reports indicating that the adipogenic activity of des-acyl ghrelin is mediated by the presence of GHSR1a in rats, suggesting the extracellular activity of the GOAT enzyme [43]. Similarly, the presence of GOAT-mediated re-acylation of exogenous des-acyl ghrelin n was reported in mouse hippocampus [44]. This is consistent with earlier reports that indicated that acyl-ghrelin is subjected to unidirectional transport through the blood–brain barrier (BBB) in mice. Mouse acyl-ghrelin transport is effective only in a brain-to-blood direction and is negligible in the opposing blood-to-brain influx, while des-acyl mouse ghrelin is subjected to passive membrane-diffusion-based transport. As acyl-ghrelin is readily detectable in mice brains, these findings indirectly suggest the presence of local extracellular GOAT activity [45]. Indeed, it was demonstrated recently that GOAT is expressed on the surface of LNCaP and 22Rv1 prostate cancer cell lines and is able to bind and modify exogenous des-acyl ghrelin [46]. The current model of potential extracellular GOAT activity postulates that GOAT is present within the cell membrane, where it binds and modifies exogenous des-acyl ghrelin, which is then recognized by the adjacent GHSR1a, enabling signal transduction into the target cell.

GOAT catalyzes the attachment of octanoic acid to ghrelin, enabling its biological activation. Its full structure remains unresolved, posing a main hindrance in the development of the next generation of selective GOAT inhibitors. The available computational model suggests that GOAT is an ER-embedded enzyme that recruits octanoyl-CoA from the cytoplasmic side to acylate-lumen-delivered ghrelin. Recent findings indicate possible extracellular GOAT activity, suggesting that DAG may be locally re-acylated at the cell surface; however, these observations are often indirect and remain controversial, as they likely do not explain the full spectrum of DAG activity.

## 4. De-Acylation and Stability of Ghrelin in Serum

Des-acyl ghrelin (DAG), the predominant form of ghrelin in the bloodstream, comprises approximately 90% of total circulating ghrelin [47]. While ghrelin acylation is crucial for its complete activity through GHSR1a [48], research indicates that DAG, despite exhibiting weak yet complete agonism, shows low potency in displacing ligand binding at GHSR1a [39]. Significant differences in potency have been observed: DAG has an EC_50_ ranging from 1.6 to 2.4 µM compared to ghrelin’s EC50 of 2–2.6 nM, spanning three orders of magnitude [40,49]. Circulating levels of ghrelin and DAG range from 0.1 to 0.5 nmol/L, although assays detecting bound peptides show higher levels (3–4 nmol/L). Therefore, DAG levels in the bloodstream are much lower than the concentration needed to activate GHSR1a [50].

Nevertheless, by activating the survival-promoting extracellular signal-regulated kinase 1/2 (ERK1/2) and PI3K/Akt signaling pathways, DAG has been demonstrated to support adipogenesis and have an anti-apoptotic impact on cardiomyocytes [43,51]. DAG was also reported to mediate anxiety-like behavior [52] and shift adipose tissue residual macrophages to M2 in mice [53]. In addition, DAG has been reported to reduce alcohol intake in rats in a dopamine-dependent manner [54]. In contrast to the postulated function of DAG, AG appears to be important in controlling autophagy, a cellular process that breaks down proteins and organelles [55,56,57]. Des-acyl ghrelin also induces food intake via orexin neurons independently of GHSR1a in mice and rats [58], although contrasting data were also reported [59]. It is worth mentioning that, in recent years, a circular peptide derivative based on the DAG_(6–13)_ sequence fragment was developed and was found to be effective in hyperphagia management in Prader–Willi syndrome patients [60,61,62].

The mechanism of DAG activity remains controversial, with some reports postulating the presence of a yet-unidentified DAG receptor [53,54,58,62,63]; others indicate an interaction between the linear DAG_(6–13)_ fragment and cell-surface proteoglycans [64], while others indicate GOAT-mediated DAG re-acylation in vivo [43,44,65]. Recent in vitro reports suggest the effect of a low (3 nM) concentration of DAG on *GHSR1a* expression, which was blocked by [D-Lys3]-GHRP-6 GHSR1a antagonist [66]. This observation seems to be in line with the potential re-acylation of DAG and its subsequent binding to ghrelin receptors. Interestingly, as both AG and DAG are present in circulation, the re-acylation of DAG would allow for the integration of total serum ghrelin into the physiological response and for the multi-phase time–response curve in GHSR1a/GOAT-co-expressing tissues, e.g., in the hippocampus, where DAG re-acylation has been demonstrated [44]. It has been suggested that, in obese T2D patients, UAG may exert its actions in a receptor-independent way by decreasing the AG/DAG ratio and modulation of the systemic AG concentration, attributing the DAG effect to the integration of total ghrelin levels, at least in part [67,68,69]. It is worth highlighting that the majority of the studies demonstrating DAG activity come from rodents, indicating potential interspecies differences in the functionality of the ghrelin system [70,71,72], or involve high pharmaceutical doses of this peptide, far exceeding physiological levels [67,68]. Some recent reports suggest the potential for DAG to modulate GLP-1R expression and GLP-1 release in a metabolic-associated fatty liver disease rat model [73,74]. Nonetheless, the main subject of controversy between these AG/DAG studies relates to the technical aspects of the sample collection methods used. Acylated ghrelin is swiftly cleared from plasma, with a half-life of 9–13 min, whereas total ghrelin (including DAG) persists for 27–34 min [75]. Early reports indicated that the ratio of active ghrelin to total ghrelin was approximately 1:20, as measured in healthy human sera by polyclonal rabbit antibodies raised against an N-terminal fragment of ghrelin (active) and C-terminal fragment (total), respectively [76]. Similar findings were reported in rat serum, where early reports indicated a 1:5 ratio of active to total ghrelin [77,78]. The presence of des-acyl ghrelin was attributed to the serum-mediated degradation of the active molecule, not to the production and release of the des-acyl molecule. Indeed, after 240 min of acyl ghrelin incubation with human serum, nearly 50% of the peptide was converted to its des-acyl form, and no further processing was observed [79,80,81]. A more pronounced effect was observed in rat serum, as already after 30 min, ~60% of the ghrelin-derived peptide was converted to its des-acyl form [79]. This activity was attributed to butyrylcholinesterase and possibly other esterases in human serum, whereas in rat serum, only carboxylesterase was involved [79,80,82]. The proteolytic degradation of ghrelin was not observed in serum, but was observed in the tissue homogenates of the stomach, liver, and kidneys, which led to the generation of the biologically inactive fragments, indicating that proteases may be involved in ghrelin processing locally, rather than systemically [79]. In parallel, other activities were identified, including the description of Acyl-Protein Thioesterase 1/Lysophospholipase (APT1) as a ghrelin-deacylating enzyme in rat stomach and in fetal bovine serum [83,84]. Unexpectedly, α_2_-macroglobulin hydrolase activity was identified in rat serum using an active-site labeling approach with a ghrelin-derived activity-based probe (ABP), potentially accounting for up to 50% of ghrelin deacylase activity [85].

These findings led to investigations into the appropriate conditions for the optimal collection and storage of samples for subsequent ghrelin detection. Indeed, some level of protection was observed after the addition of PMSF to serum samples [79] or after the addition of EDTA and aprotinin during sample collection [86]. Also, the acidification of the serum samples with HCl at pH 3–4 led to the significant protection of ghrelin, an observation consistent with the inhibition of serum esterases by low pH [86]; however, increased ghrelin deacylation was observed in the samples containing ≥100 mM HCl [81]. Further research indicated 4-(2-aminoethyl)benzenesulfonyl fluoride hydrochloride (AEBSF) to be an effective compound for active ghrelin protection in human serum samples [81]. In recent years, alkyl fluorophosphonate inhibitors were proposed to be the optimal compounds for stabilizing ghrelin octanoylation in biological samples. This is exemplified by AG protection in the presence of methoxy arachidonyl fluorophosphonate (MAFP) in biological samples, including cell lysates and rat blood, a treatment superior to all previously described treatments [87]. Indeed, the addition of MAFP during the collection of the rat blood samples resulted in unprecedented acyl-ghrelin protection and led to the detection of unexpectedly high active ghrelin levels [87]. It is worth noting that significant levels of ghrelin deacylase APT1 were released from the mouse-derived RAW264.7 macrophage cell line upon LPS stimulation, and similar enzymes were identified in fetal bovine serum (FBS) [84], indicating the potential need for ghrelin stabilization, even in cell-culture-based assays. Indeed, it has been suggested that the inherent instability of ghrelin in serum may lead to the significant underestimation of active/total ghrelin ratios, especially in earlier reports, where no protection procedures were employed [87]. Unfortunately, the lack of a standardized protocol for sample collection in these ghrelin studies often hindered the correct interpretation of the results and prohibits direct comparisons of different studies, which remains a significant problem in the field [68,69].

DAG, which constitutes about 90% of total circulating ghrelin, exhibits a markedly lower potency than AG when interacting with the GHSR1a receptor. Nonetheless, DAG activates survival and metabolic pathways such as ERK1/2 and PI3K/Akt. Its physiological role remains controversial, with evidence suggesting receptor-independent actions, extracellular re-acylation by GOAT, or interaction with yet-unidentified receptors. Methodological issues, particularly rapid ghrelin deacylation and inconsistent sample treatment between different studies, complicate the interpretation of DAG’s biological significance and may, at least in part, underlie the conflicting findings seen across studies.

## 5. Physiological Roles of Ghrelin

**Figure 2:**
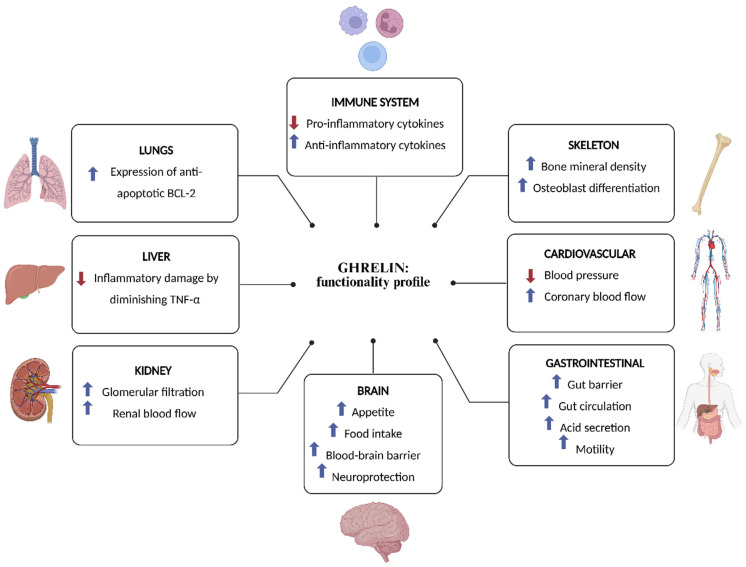
Functionality profile of ghrelin. Schematic illustration of the hormonal actions of ghrelin in different target organs and tissues. Red arrows indicate decrease, while blue arrows indicate increase. Created in BioRender. Kantyka, T. (2025) https://BioRender.com/6s2oynh.

Ghrelin has emerged as a pivotal factor in numerous physiological functions (Figure 2). Their functional scope encompasses growth hormone (GH) secretion, stimulation of appetite and food intake, maintenance of glucose homeostasis, reproductive system effects, modulation of gastric secretion and gastrointestinal motility, improvements in gut barrier function and circulation, and immunomodulatory effects by downregulation of pro-inflammatory and upregulation of anti-inflammatory cytokines [1,88,89]. In addition, ghrelin enhances neuroprotection, contributes to cardiovascular functions such as lowering blood pressure and enhancing coronary blood flow, influences sleep/wake rhythms, increases the expression of anti-apoptotic BCL-2 in lung cells, enhances renal blood flow and promotes glomerular filtration in the kidneys, and stimulates osteoblast proliferation and bone formation [90,91,92,93,94,95].

### 5.1. Appetite and Energy Homeostasis

Ghrelin earned its nickname as the “hunger hormone” because it stimulates appetite by sending signals to the brain to indicate that it is time to eat, thereby increasing food intake and promoting fat storage [96]. Ghrelin levels in serum increase before meals and decrease afterward [7,13]. Ghrelin plays a role in short-term food intake regulation and long-term body weight control by reducing fat utilization [97]. This effect on feeding is facilitated through GHSR1a, evidenced by the absence of its orexigenic impact in knockout mice [97].

By regulating appetite, ghrelin acts as a key regulator of energy homeostasis integrating signals from peripheral nutritional status to the central nervous system. Specifically, ghrelin promotes increased food consumption and fat storage while potentially slowing overall metabolic rate and reducing the body’s capacity to burn fat, which helps conserve energy during periods of fasting or low nutrient availability [98]. In terms of energy balance, ghrelin contributes to efficient metabolic adaptations by modulating energy expenditure; for instance, it can induce changes that favor energy conservation, such as altering thermogenesis or substrate utilization in tissues like adipose and muscle. It also stimulates the release of growth hormones from the pituitary gland, which indirectly supports metabolic processes like protein synthesis, lipolysis, and glucose regulation [99]. Regarding glucose metabolism, ghrelin influences insulin secretion and sensitivity in a GLP-1 opposing and circadian rhythm-related fashion [100], often promoting a state that maintains blood sugar levels during fasting, although chronic elevations may contribute to insulin resistance in certain contexts. Additionally, ghrelin exerts control over the lipid metabolism by regulating the central and peripheral pathways, including the promotion of lipogenesis in the liver and adipose tissue, which aids in energy storage [101]. Emerging research suggests that it may also play a protective role in preventing excessive obesity and insulin resistance during growth phases, such as in catch-up growth scenarios, by balancing the energy metabolism with anabolic processes [102]. However, studies on ghrelin knockout models indicate that, while it is important, its role in appetite and metabolism may be somewhat redundant, as animals lacking ghrelin do not always show significant reductions in food intake or metabolic disruptions [103]. Overall, ghrelin’s metabolic effects are context-dependent, varying with factors like nutritional status, circadian rhythm, and interactions with other hormones such as leptin [104].

In the innate immune system, ghrelin’s impact on macrophage polarization directly ties to metabolic reprogramming. Macrophage diversity is described by the outdated M1/M2 model, created more than 20 years ago, which distinguishes between M1, or “classically” activated macrophages, and M2, or “alternatively” activated macrophages, based on the effect of in vitro macrophage stimulation with type 1 or type 2 cytokines [105]. In the more recent version, the “M1-like” phenotype is usually described as pro-inflammatory and is induced by Toll-like receptor (TLR) ligands and type 1 cytokines, namely IFN-γ and TNF-α; ‘M2-like’ macrophages, having anti-inflammatory properties, are activated by IL-4 or IL-13 and produce TGF-β. This nomenclature, although oversimplified, is widely used today [106,107]. Favoring an M2-like state that relies on oxidative phosphorylation and fatty acid oxidation (FAO) phenotypes involves the activation of AMP-activated protein kinase (AMPK) and peroxisome proliferator-activated receptor gamma (PPARγ). In LPS-stimulated macrophages, exogenous ghrelin promotes FAO and oxidative metabolism, as PPARγ regulates lipid handling and cholesterol efflux (CD36 receptor), reducing foam cell formation and supporting tissue repair [108]. Ghrelin also improves mitochondrial fitness, enhancing bioenergetic efficiency, restructuring mitochondrial networks, and reducing oxidative stress, which aids macrophage survival and function under inflammatory stress [109]. Contrasting evidence from metabolic disorders shows ghrelin signaling via GHSR1a drives M1 polarization. This remodels metabolism toward increased glycolysis and decreased FAO, mediated by the PKA-CREB-IRS2-AKT2 pathway, which activates NF-κB nuclear translocation [110]. In aging or high-fat-diet models, GHSR1a expression rises, which is coupled with pro-inflammatory cytokine expression (e.g., TNF-α, IL-1β, MCP1) and macrophage infiltration into adipose tissue. GHSR1a ablation shifts toward M2, increasing norepinephrine production and enhancing lipolysis/thermogenesis in adipose tissues [111]. In fructose-exposed macrophages, GHSR1a mediates inflammation via CREB-AKT-NF-κB, upregulating fructose uptake (via GLUT5) and metabolism (via ketohexokinase and AMPK-AKT/p38), creating a pro-inflammatory feedback loop [112]. Ghrelin’s nutrient-sensing role via GHSR1a links it to macrophage metabolic reprogramming. In anti-inflammatory contexts, it supports efficient energy production, potentially via AMPK-driven catabolism and mitochondrial optimization, improving cell welfare and reducing apoptosis (e.g., in LPS-induced acute respiratory distress) [93]. In pro-inflammatory settings, it favors Warburg-like glycolysis for rapid energy access in M1 cells, contributing to meta-inflammation in obesity or aging [110]. Ghrelin influences cholesterol handling and lipogenesis. It upregulates sterol transporters for efflux, inhibiting lipid accumulation in macrophages, which ties to anti-inflammatory effects [108]. However, in chronic conditions, GHSR1a deficiency reduces lipid-associated macrophages, alleviating inflammation. Pro-inflammatory effects exacerbate chronic conditions like adipose inflammation, but may aid in acute nutrient-sensing responses. Ghrelin’s effects on mitochondrial fitness are particularly evident in models of chronic kidney disease (CKD), aging, and cachexia, where it promotes biogenesis, oxidative capacity, and overall metabolic health. In CKD models, ghrelin normalizes impaired mitochondrial oxidative capacity by restoring activities of enzymes like cytochrome c oxidase and citrate synthase. This is linked to the upregulated expression of biogenesis regulators (e.g., PGC-1α, PGC-1β, mitochondrial transcription factor A [mtTFA]) and lipid metabolism factors (e.g., PPARα). It also reduces elevated muscle triglycerides, favoring lipid oxidation and insulin signaling via AKT phosphorylation, especially when combined with increased food intake [113]. In aging muscle, ghrelin treatment recovers declining mitochondrial activity and exercise endurance. This involves the dual activation of TORC1 (promoting protein synthesis) and AMPK (enhancing energy sensing and mitochondrial function), leading to increased mitochondrial abundance and muscle mass [114]. Unacylated ghrelin protects against muscle wasting and mitochondrial dysfunction in cancer cachexia models, preserving neuromuscular integrity and countering tumor-induced metabolic decline [115]. Conversely, ablating the ghrelin receptor in aging models improves mitochondrial function independently of ghrelin presence: GHSR1a knockout mice show elevated PGC-1α and UCP3 (markers of biogenesis and uncoupling), enhanced AMPK and ACC phosphorylation (supporting β-oxidation), reduced lipid accumulation, and better insulin sensitivity via GLUT4 and IRS1. This is potentially mediated by increased irisin (from FNDC5), promoting a functional mitochondrial profile and mitigating age-related metabolic decline [116]. Ghrelin exerts significant cardioprotective effects against conditions such as ischemia/reperfusion injury, myocardial infarction, heart failure, and drug-induced cardiotoxicity [91,117]. Key aspects of ghrelin’s cardioprotection include its ability to inhibit apoptosis and improve mitochondrial fitness, which are interconnected mechanisms critical for maintaining cardiomyocyte viability under stress. Apoptosis in cardiomyocytes is a major contributor to cardiac damage during pathological states like oxidative stress, hypoxia, or toxin exposure, often involving the intrinsic (mitochondrial) pathway where pro-apoptotic factors like Bax trigger cytochrome c release from mitochondria, leading to caspase activation and cell death [118]. Ghrelin counters this by downregulating pro-apoptotic proteins such as Bax and cleaved caspases (e.g., caspase-3, -8, -9) while upregulating anti-apoptotic proteins like Bcl-2, thereby shifting the balance toward cell survival. Ghrelin safeguards cardiac mitochondrial function during myocardial ischemia–reperfusion, a common model of heart attack. In rat models, pretreatment with ghrelin restores ATP levels, reduces malondialdehyde (MDA, a marker of oxidative stress), and preserves mitochondrial ultrastructure [119]. This protection stabilizes energy metabolism and prevents cellular damage. Activation of mitochondrial ATP-sensitive potassium (mitoKATP) channels maintains mitochondrial ATP and connexin 43 (Cx43) expression in mitochondrial fractions. Blocking ghrelin receptor with DLys3-GHRP-6 reverses these benefits, confirming receptor dependency. In aged rat hearts (20–22 months) subjected to ishemia–reperfusion, preconditioning mesenchymal stem cells (MSCs) with ghrelin before intra-myocardial injection provides superior cardioprotection by boosting mitochondrial bioenergetics [120]. This reduces infarct size, cardiotroponin release, and improves left ventricular function. Ghrelin also restores ishemia–reperfusion-induced mitochondrial membrane potential depolarization, elevates ATP production, and normalizes reactive oxygen species (ROS) levels. This mechanism is based on an enhanced autophagy flux (downregulates Beclin-1 and P62, upregulates LC3-II and LC3-II/LC3-I ratio), which clears damaged mitochondria (mitophagy). Inhibiting autophagy with chloroquine abolishes these effects. Synergy with nicotinamide mononucleotide (NMN) further amplifies mitochondrial rescue via the autophagy/mitochondrial pathways. Ghrelin emerges as a potential therapy for heart failure by improving mitochondrial function in cardiac tissue, often via GH and insulin-like growth factor-1 (IGF-1) signaling. In rat models (e.g., post-coronary ligation or doxorubicin-induced), ghrelin preserves mitochondrial energy metabolism, reduces inflammation/remodeling, and enhances contractility without calcium overload [121]. In vitro studies using oxidative stress (H_2_O_2_) yield context-dependent results. In neonatal rat ventricular myocytes (NRVMs), ghrelin overexpression (via lentiviral vectors) improves viability against H_2_O_2_, requiring prohormone convertase 1/3 (PCSK1) for mature ghrelin processing [122]. However, in H9c2 cells (lacking PCSK1), no protection occurs, and mitochondrial membrane potential declines without recovery. In human iPSC-derived cardiomyocytes, no benefits are seen despite the presence of PCSK1, suggesting cell-type specificity. Overall, ghrelin’s cardioprotective role in cardiac mitochondria is evident in stress paradigms, promoting bioenergetic efficiency, reducing ROS/oxidative damage, and enhancing mitophagy/apoptosis resistance. These mechanisms often involve the GHSR1a receptor, mitoKATP, autophagy, and PI3K-Akt signaling. These findings support ghrelin’s therapeutic potential in CVD, although human trials are limited and the effects may depend on acylation status, dosage, and cellular context. Further research is needed on direct mitochondrial-targeted interventions.

Overall, while many studies portray ghrelin as having anti-inflammatory and metabolism-supporting effects, chronic metabolic contexts suggest its pro-inflammatory role, at least to some extent. This duality may stem from acylated vs. des-acylated ghrelin forms, dosage, or environmental cues. Further research, including cell-specific knockouts, is needed to clarify the mechanisms.

### 5.2. Ghrelin as an Anti-Inflammatory Agent

Ghrelin is recognized for its broad anti-inflammatory effects across multiple physiological systems. In the central nervous system, AG reduces neuronal damage following subarachnoid hemorrhage in rats and mitigates neuroinflammation in Alzheimer’s disease models [123]. Ghrelin’s immunoregulatory role is exemplified by its suppression of pro-inflammatory cytokine production. Notably, in aged septic rats, both ghrelin and GH attenuate immunosuppression via vagus the nerve-dependent inhibition of transforming growth factor-beta (TGF-β) production [124].

In the gastrointestinal tract, ghrelin demonstrates therapeutic potential in relieving colitis, while also exerting beneficial effects in metabolic disorders such as type 2 diabetes [62,125]. Beyond these roles, ghrelin influences skeletal muscle and cardiovascular and respiratory functions [126,127].

In a murine model of elastase-induced emphysema, ghrelin treatment attenuated pulmonary inflammation, promoted macrophage polarization toward the M2 phenotype, reduced collagen deposition, and increased elastic fiber content, collectively contributing to improved alveolar architecture and lung function. Additionally, ghrelin improved cardiovascular dysfunction and increased both lean and total body mass [127].

Consistently, ghrelin has been shown to reduce inflammation and disease severity in numerous conditions, including sepsis, inflammatory bowel disease, arthritis, pancreatitis, obesity, autoimmune encephalomyelitis, and diabetic nephropathy [123,128,129,130]. In turn, this underscores the evident link between ghrelin and chronic diseases. Studies from many laboratories collectively provide robust evidence that ghrelin exerts anti-inflammatory effects in human monocytes and macrophages by reducing pro-inflammatory cytokine production, inhibiting inflammatory signaling pathways, promoting anti-inflammatory macrophage polarization, and decreasing adhesion and oxidative stress [131,132,133,134].

Ghrelin hinders the generation of pro-inflammatory cytokines from monocytes, T-cells, and macrophages [131]. It also hampers the leptin-triggered expression of pro-inflammatory cytokines such as IL-1β, IL-6, and TNF-α while boosting the production of anti-inflammatory cytokines like TGF-β and IL-10 [89,132]. HMGB1 (High-Mobility Group Box 1) is a nuclear protein produced by the *HMGB1* gene, which stabilizes chromosome structure and regulates gene expression [135] and, when translocated to the cytoplasm, induces autophagy [136]. In immune cells, HMGB1 is directed for secretion [137] and acts as an alarmin, recognized by RAGE- and TLR-family receptors [138]. It is secreted by diverse immune cells, including monocytes, macrophages, and dendritic cells, prompting the release of pro-inflammatory cytokines like TNF-α, IL-1, IL-6, and IL-8, thus intensifying inflammation [139,140]. Ghrelin treatment impedes HMGB1 translocation from the nucleus to the cytoplasm, reducing its secretion and thereby dampening inflammation [131]. The delayed release of ghrelin by macrophages when stimulated by substances like LPS, IL-1, and TNF-α may act as a regulatory mechanism to avert excessive inflammation [141].

Remarkably, GHSR deletion mutants (Ghsr^mutant^) of macrophages and microglia reduce their inflammatory responses to fructose, indicating that GHSR mediates fructose-induced inflammation. Furthermore, GHSR regulates fructose transport and metabolism and mediates fructose-induced inflammatory activation through the CREB–AKT–NF-κB and p38 MAPK signaling pathways [112]. These findings reveal that the nutrient-sensing receptor GHSR plays a crucial role in fructose-mediated inflammatory activation. Interestingly, at the molecular level, GHSR metabolically programs macrophage polarization through the PKA–CREB–IRS2–AKT2 signaling pathway [110], an axis which is not characteristic for other cell types. Insulin signaling is a master regulatory pathway of the metabolism and is involved in macrophage polarization [142]. Insulin receptor deficiency in macrophages protects against inflammation [143,144,145], and IRS2 deletion promotes IL-4-induced M2 macrophage polarization [146]. Thus, nutrient-sensing ghrelin signaling is linked to immune regulation and meta-inflammation.

Obesity-related chronic inflammation or meta-inflammation has been linked to a wide range of metabolic dysfunctions such as insulin resistance [147,148], fatty acid dysregulation [149,150], and non-alcoholic fatty liver disease (NAFLD) [151,152]. Macrophages are among the first responders of inflammation in response to metabolic dysregulation [153], and energy balance plays a major role in their responses, as discussed above. Meta-inflammation through cytokines and other inflammatory mediators promotes macrophage recruitment and activation in tissues, leading to deleterious effects in, for example, adipose tissue and liver [154,155,156]. Ghrelin is known to stimulate appetite and promote obesity/insulin resistance via the G protein-coupled receptor (GPCR) [48,157,158,159,160]. GHSR1a expression is very high in macrophages and monocytes [111,131]. In mouse peritoneal macrophages, expression reaches 60% relative to the hypothalamus, the tissue with the highest expression [111]. The global ablation of GHSR1a promotes anti-inflammatory change in peritoneal macrophages and adipose tissue macrophages in aging mice [111]. Global GHSR1a ablation has also been shown to alleviate adipose tissue inflammation and non-alcoholic steatohepatitis (NASH) induced by high-fructose corn syrup (HFCS) [161]. Adipose tissue macrophages of such mice show a reduced expression of pro-inflammatory markers (M1), including monocyte chemoattractant protein-1 (MCP-1), TNF-α, and inducible nitric oxide synthase (iNOS), while the anti-inflammatory markers arginase-1 (Arg-1) and macrophage galactose-type lectin-1 (Mgl-1) are increased [162]. In vitro studies have also shown that antagonist or knock-down (siRNA) of GHSR1a decreases the expression of pro-inflammatory cytokine genes in the LPS-stimulated macrophage cell line RAW264.7 [111,161]. These observations suggest that GHSR1a has a cell-autonomous effect in macrophages, and that GHSR1a probably plays a key role in macrophage programming. In myeloid-specific GHSR1a, knockout mice tissue analysis shows significantly decreased monocyte/macrophage infiltration, pro-inflammatory activation, and lipid accumulation. Ex vivo, GHSR1a-deficient macrophages were protected against palmitic acid (PA)- or LPS-induced pro-inflammatory polarization, showing reduced glycolysis, increased fatty acid oxidation, and decreased NF-κB nuclear translocation [110]. As a G protein-coupled receptor, GHSR has a high ligand-independent constitutive activity of ~50% of its maximal activity [163,164,165]. Thus, the effect of GHSR1a in macrophages may be mediated by both ligand-dependent and -independent (constitutive) mechanisms. The global deletion of GHSR1a does not alter serum ghrelin levels compared to wild-type mice under both fed and fasted conditions [158]. Although evidence in primary human macrophages remains limited, it is speculated that, even without endogenous ghrelin, GHSR1a may modulate macrophages by altering downstream signaling pathways. Most evidence comes from animal models (e.g., murine macrophages) or indirect studies in human cell lines (e.g., THP-1 macrophages), which may not reflect primary human macrophage behavior. There is no direct evidence in human macrophages, suggesting that GHSR1a’s constitutive activity promotes pro-inflammatory responses. Constitutive activity alone does not appear to drive pathways like NF-κB activation or pro-inflammatory cytokine release in human macrophages [166,167]. Thus, we speculate that the potential inflammatory outcome of GHSR1a’s constitutive activity may depend on the macrophage’s activation state (e.g., resting, M1, or M2) and the microenvironment. For instance, in resting human macrophages, constitutive activity likely maintains an anti-inflammatory tone, while in highly inflamed states, its impact might be overshadowed by dominant pro-inflammatory signals (e.g., LPS-induced TLR4 activation).

Ghrelin exerts potent anti-inflammatory and immunoregulatory effects by overwhelmingly suppressing pro-inflammatory cytokines and skimming macrophage polarization towards the M2 phenotype. Ghrelin limits NF-κB and MAPK activation, enhances anti-inflammatory cytokine release, and maintains cellular fitness, integrating nutrient sensing and immune homeostasis. However, in chronic metabolic disorders, this same signaling axis can promote pro-inflammatory macrophage activity, illustrating ghrelin’s complex role in inflammation and metabolic regulation.

### 5.3. Ghrelin Is a Cardioprotective Factor

Ghrelin has recently emerged as important regulator of cardiovascular homeostasis and myocardial stability [168]. Experimental and clinical evidence indicates that both acylated (AG) and des-acyl ghrelin (DAG) play important roles in the pathogenesis and modulation of structural heart diseases [169]. The identification of ghrelin and GHSR1a mRNA in the myocardium of humans and experimental animals suggests a role of the ghrelin system in the heart [170,171]. Numerous studies have demonstrated the cardioprotective effects of endogenous and exogenous ghrelin in diverse cardiac conditions, including myocardial infarction (MI), ischemia/reperfusion injury (IRI), drug-induced cardiac injury, and heart failure (HF) [168,172,173,174]. These activities were at least partially related to the anti-inflammatory effects of ghrelin. For example, in a rat doxorubicin-induced cardiac cell death model, the protective effects of ghrelin were attributed to the normalization of IL-6/JAK2/STAT3 signaling, increase in ERK1/2 activation, and inhibition of STAT1 [174]. Similarly, in a recent work on IRI mice conducted in a heterotopic cervical heart transplantation model, ghrelin exerted a beneficial role, which was attributed to a reduction in inflammatory response and apoptosis inhibition [172]. These results are also reflected in populations of human patients, as, in a recent human trial, the effects of ghrelin were assessed in patients with heart failure and reduced ejection fraction [173]. This study found that AG increased cardiac output without causing hypotension, tachycardia, arrhythmia, or ischemia in patients. At the same time, in isolated mouse cardiomyocytes, AG increased contractility and did not induce Ca^2+^ mobilization [173].

One of the earliest recognized effects of ghrelin was vasodilatation, which occurs via endothelium-dependent mechanisms involving activation of the PI3K–AKT–eNOS pathway and increased nitric oxide (NO) production [175,176,177,178]. Ghrelin improves endothelial-dependent relaxation and counteracts oxidative-stress-induced vascular dysfunction. In human studies, intravenous ghrelin infusion reduced muscle sympathetic nerve activity and blunted cardiovascular stress responses in both lean and overweight/obese male individuals [50], indicating its role in both the peripheral and central modulation of cardiovascular response.

Functionally, ghrelin acts through GHSR1a-dependent and -independent mechanisms to influence cardiomyocyte metabolism, contractility, and survival. It has been shown to attenuate ventricular remodeling, limit fibrosis, inhibit apoptosis, and enhance endothelial function, thereby maintaining myocardial structure and performance under stress. These protective mechanisms involve the activation of pro-survival signaling pathways, reduction in oxidative stress, modulation of the autonomic nervous system, and suppression of inflammatory cascades [168,170,172,173,174,179,180,181].

At the endothelial level, ghrelin protects cells from apoptosis and oxidative damage, enhancing survival under hyperglycemic or inflammatory stress [71,177,182]. This endothelial-protective role positions ghrelin as a physiological factor that maintains vascular integrity under metabolic burden. Experimental studies consistently report that ghrelin restores NO bioavailability, decreases reactive oxygen species, and inhibits endothelial senescence, thereby contributing to vascular homeostasis [175,176,177,178].

In the myocardium, ghrelin exhibits anti-apoptotic, anti-fibrotic, and contractility-preserving properties. In a model of doxorubicin-induced cardiotoxicity, Pei et al. (2014) [51] demonstrated that des-acyl ghrelin protects against cardiomyocyte apoptosis and fibrosis through GHSR1a-independent mechanisms, as concomitant DAG/[d-Lys3]-GHRP-6 treatment did not affect the observed levels of protection. This effect was linked to the activation of AKT/ERK pathways and the inhibition of mitochondrial oxidative stress [51]. More recently, AG was found to limit DOX-induced cardiac toxicity in rats by the activation of ERK1/2 and JAK2/STAT3 and inhibition of STAT1 [174].

In obesity-related cardiac injury, ghrelin functions as a protective factor mitigating lipotoxic and inflammatory stress. It was demonstrated that, in a mouse model of obesity and in a palmitic acid (PA)-treated cardiomyocyte cell line, ghrelin treatment and/or overexpression activated the complementary H19/miR-29a/IGF-1 pathway, contributing to its cardioprotective action [183]. More recent reports show that ghrelin attenuates obesity-induced myocardial damage through the lncRNA-HOTAIR/miR-196b axis [184]. These findings link the role of ghrelin with the non-coding, miRNA-mediated regulation of cardiac gene expression.

Recent data emphasize the interaction between ghrelin and its endogenous antagonist, LEAP-2. Recently, it was reported that knockdown of LEAP-2 ameliorated HFD-induced hyperlipidemia, inflammation, and myocardial injury. This effect was accompanied by limitation of the inflammatory responses, as macrophage infiltration and enhanced M2 polarization were attributed to an improved GHSR1a–ghrelin interaction in the absence of LEAP-2 [185].

Not surprisingly, ghrelin is well positioned in the management of the inflammation-induced cardiovascular damage. In an elastase-induced emphysema model, ghrelin administration improved both pulmonary and cardiovascular performance [127], underscoring its integrative protective role in the control of systemic inflammation. This indicates the translational potential of the pharmaceutic regulation of ghrelin system in the management of cardiovascular diseases [169,186]. Nonetheless, the translation of these findings needs to be taken with care, as there are some reported cases of adverse cardiac effects upon anamorelin treatment in cachexia patients, most likely related to impaired liver function [187,188], and an ibutamoren phase IIb study in elderly hip fracture patients was terminated due to early symptoms of congestive heart failure [189]. These alarming observations indicate the risks related to the application of broad-spectrum agents in sensitive populations.

Collectively, current evidence supports ghrelin as a multifaceted regulator of cardiovascular health, integrating metabolic and anti-inflammatory responses with vascular and myocardial protection. However, the relative roles of acylated versus des-acyl ghrelin, receptor-independent pathways, and long-term clinical implications remain incompletely defined, and are open to future investigations. Nonetheless, recent findings, which unveil biased GHSR1a signaling and provide a structural template for the development of more selective compounds, allow for cautious optimism regarding the development of future therapeutics targeting individual aspects of ghrelin’s biology.

### 5.4. Ghrelin as a Regulator of Fertility

As an orexigenic hormone that signals nutrient insufficiency, ghrelin functions as a key metabolic messenger linking energy status with reproductive function [190]. In women, ghrelin acts predominantly as a negative modulator of fertility, particularly under conditions of caloric restriction or excessive energy expenditure [191,192,193]. Circulating ghrelin levels increase with age and throughout the menopausal transition [194,195], potentially contributing to postmenopausal metabolic alterations and an increase in adverse cardiovascular risk factors [195]. Elevated ghrelin concentrations have been reported in women suffering from functional hypothalamic amenorrhea associated with anorexia nervosa or intense physical training. Ghrelin was postulated to act as a restraining metabolic signal preventing the restoration of cyclicity in women, prolonging amenorrhea [193].

Physiologically, circulating ghrelin levels increase in newborns, reaching a peak during early childhood and decreasing until the end of puberty in correlation with early growth and reproductive maturation [196]. A recent study on male rats found a correlation between caloric restriction and increased ghrelin levels, which were implicated in the disruption of the hypothalamic-pituitary-gonadal (HPG) axis. The observed effects included reduced levels of follicle-stimulating hormone (FSH), luteinizing hormone (LH), and testosterone, indicating a negative impact on fertility, libido, semen quality, and overall sexual function [197]. Administration of exogenous ghrelin in prepubertal male rats delayed the onset of puberty, suppressing serum LH and testosterone levels and limiting the number of pups per litter in pregnant females [198]. In addition, gonadotropin-releasing hormone (GnRH) and LH secretion were negatively affected by ghrelin injections in female rats [199]. At the same time, ghrelin stimulated basal LH and FSH secretion by pituitary tissue in vitro, concurrently inhibiting GnRH-stimulated LH release in the same in vitro system. These results suggest the complex, context-dependent, and tissue-specific action of ghrelin on the gonadotropic axis, with predominant inhibitory effects at the central level [199,200], which varies with sex, developmental stage, and hormonal milieu [198,199,201]. In female mammals, chronic or high-dose ghrelin treatment reduces ovulation, pregnancy rates, and embryo implantation, while also impairing embryonic development in vitro and murine models [202,203,204]. Interestingly, ghrelin response was bell-shaped in ovine models, and ghrelin antagonist D-Lys3 also elicited negative outcomes, indicating that an adequate concentration of ghrelin may be required during pregnancy [203,205]. Indeed, ghrelin antagonism impaired fetal implantation, potentially in relation to the increased inflammatory state markers [206]. In pregnant rats, ghrelin crosses the placental barrier and increases pup birth weight [207]. In humans, ghrelin was indicated as an important factor impacting decidualization of human endometrial stromal cells and embryo implantation [208].

GHSR1a expression was identified in the hypothalamus regions responsible for reproductive regulation [209,210], including Kiss1 neurons [211,212] and pituitary gonadotrophs [213], and these expression patterns were sex-dependent and susceptible to estrogen regulation. Although GnRH neurons themselves lack GHSR1a expression, ghrelin likely acts indirectly via inhibitory neuropeptide Y (NPY) and AgRP neurons, or through the modulation of kisspeptinergic input, to suppress GnRH release [203,212,214]. Central administration of ghrelin reduces GnRH secretion and decreases pulse frequency, without affecting pulse amplitude, leading to decreased luteinizing hormone (LH) and/or follicle-stimulating hormone (FSH) levels in vivo [199,214,215,216,217,218]. This effect was at least partially attributed to the fact that ghrelin treatment decreased kisspeptin (Kiss1) production [214], an essential stimulator of the HPG axis, which controls gonadotropin secretion [219].

In addition to its central effects, ghrelin also exerts direct peripheral actions on the gonads, particularly within the ovaries. The expression of ghrelin and its receptor has been detected in the testes and ovarian tissues of multiple vertebrate species, including sheep [220,221], pigs [222], rats [223], and humans [224,225]. In the ovaries, ghrelin and GHSR1a expression was localized in oocytes, corpus luteum, granulosa, and stromal cells [220,221,222,223,224,225,226]. Functionally, ghrelin lowers circulating estrogen and progesterone concentrations in female rats and decreases the expression of estrogen receptor β [227]. Similar effects were observed in female sheep, as long-term ghrelin infusion reduced levels of LH and progesterone and negatively affected FSH-induced super-ovulatory response and the number of embryos [228]. In luteal cells, ghrelin acts directly via the GHSR1a receptor to inhibit progesterone synthesis and release. In addition, ghrelin decreased VEGF production and downregulated prostaglandin PGE2 release while increasing PGF2α release, consistent with a negative influence on luteal function [229,230]. In rats, morphological studies have revealed that ghrelin administration increases the number of follicles while reducing corpus luteum formation, consistent with inhibited follicular maturation and ovulation, and suggesting ghrelin as a suppressor of the female reproductive system [231]. In contrast, GOAT-knockout mice, which lack endogenous acylated ghrelin, exhibit reduced small and primordial follicle populations, suggesting that physiological ghrelin signaling contributes to optimal ovarian maturation [232]. Recently, ghrelin and GHSR1a agonists were investigated as factors improving the maturation rate of human oocytes in vitro; however, without success, as only nuclear stages of oocyte maturation were improved by low concentrations of GHRP-6, while higher concentrations were detrimental [233].

Interestingly, the inhibition of *GHSR1a* expression by miR-128-3p was recently shown to decrease follicular granulosa cell proliferation and increase apoptosis, consistent with the pro-regenerative and anti-inflammatory role of the ghrelin–GHSR1a axis in the female reproductive tract [234].

Taken together, these findings indicate that ghrelin systemically acts as a limiting factor on reproduction, integrating central and peripheral signals to align fertility with energy status. When nutritional reserves are low, ghrelin levels increase and suppress hypothalamic and gonadal activity to limit reproductive function. Conversely, the normalization of metabolic balance restores fertility. At the same time, both infra and supraphysiologic levels can be detrimental in early pregnancy, highlighting the fine-tuned, bell-shaped response to ghrelin in female reproductive function, potentially related to inflammation control [235,236]. Thus, ghrelin serves as an endocrine mediator that couples immunity, metabolism, and reproductive capacity, ensuring reproductive success only under energetically favorable and non-inflammatory conditions [237].

### 5.5. Neurobehavioral and Neuroprotective Effects of Ghrelin

The hypothalamus is a small brain region located just below the thalamus, responsible for central regulation of appetite and energy homeostasis. Structurally, the hypothalamus consists of several distinct nuclei: the arcuate nucleus (ARC), paraventricular nucleus (PVN), lateral hypothalamic area (LHA), ventromedial nucleus (VMN), and dorsomedial nucleus (DMN). The ARC lies adjacent to the median eminence, a circumventricular organ characterized by fenestrated capillaries and an atypical blood–brain barrier (BBB). This proximity provides ARC neurons with relatively direct access to circulating hormones and nutrients without crossing the typical BBB. Owing to this unique anatomical feature, the ARC is considered a principal hypothalamic site for sensing peripheral metabolic signals. Two major neuronal populations are present in the ARC: (i) neurons co-expressing orexigenic (appetite-stimulating) neuropeptides, including neuropeptide Y (NPY) and agouti-related peptide (AgRP); and (ii) neurons expressing anorexigenic (appetite-limiting) neuropeptides, including proopiomelanocortin (POMC) and cocaine- and amphetamine-regulated transcript (CART). These populations serve as first-order integrators through which peripheral metabolic signals affecting appetite are relayed [238].

As in other organs, the function of ghrelin in the central nervous system (CNS) is mediated by activation of GHSR1a. Its expression has been identified in the pituitary gland, ARC, VMN, PVN, and, to some extent, DMN [171,239,240,241]. Ghrelin activity in hypothalamic neurons is part of the brain’s fatty acid-sensing mechanism. The binding of ghrelin to GHSR1a activates PLC–IP3/DiAcGly–PKC signaling and increases intracellular calcium levels. This, in turn, activates calcium-/calmodulin-dependent protein kinase 2 (CamKII), which phosphorylates AMP-activated protein kinase (AMPK) and promotes the formation of a stable AMPK/CaMKK2 complex. This complex increases the phosphorylation of acetyl-CoA carboxylase (ACC), lowering malonyl-CoA and thereby disinhibiting carnitine palmitoyltransferase-1 (CPT1A and CPT1C) [242,243,244,245]. CPT1A shuttles acylcarnitines, allowing for long-chain fatty acids to cross the mitochondrial membrane for subsequent β-oxidation [246,247]. An enhanced fatty acid metabolism can elevate mitochondrial reactive oxygen species (ROS) and upregulate the free-radical-scavenging uncoupling protein-2 (UCP2), which contributes to the activation of NPY/AgRP neurons and stimulation of ghrelin-induced food intake [248]. The CPT1C isoform increases ceramide levels and upregulates NPY and AgRP while downregulating POMC expression, thereby increasing NPY/AgRP and decreasing POMC activity in the ARC, effectively stimulating appetite. In addition, ghrelin modulates NPY-related neurons in the PVN and ARC, increasing the GABAergic suppression of POMC neurons, which further promotes appetite [249,250].

Beyond its classical roles in appetite regulation and pituitary signaling, ghrelin exhibits potent anti-inflammatory and neuroprotective properties [132]. Preclinical studies demonstrate that exogenous ghrelin maintains blood–brain barrier (BBB) integrity, reduces oxidative damage, and limits neuronal apoptosis after traumatic brain injury (TBI) and ischemia–reperfusion injury (IRI) [251,252,253,254]. These effects are linked to the modulation of fibroblast growth factor signaling [255] and the activation of the PI3K/Akt pathway, which promotes neuronal survival via the regulation of GSK-3β and Bcl-2 [256,257,258].

Ghrelin has been implicated in several mental disorders, including anorexia nervosa and bulimia—via appetite regulation—as well as depression, anxiety, and schizophrenia [259,260]. The ghrelin gene polymorphism Leu72Met has been associated with depression, but not with panic disorder, in patients [261].

Depression is a multifactorial disorder with genetic, neuroendocrine, immune, and metabolic influences [262,263]. Beyond monoamine deficiency [264], hypothalamus–pituitary–adrenal (HPA) axis dysregulation [265], excitatory/inhibitory imbalance [266], and neuroinflammation [267], growing evidence implicates ghrelin in mood regulation.

Ghrelin may influence depression in part through the normalization of the hypothalamic–pituitary–adrenal (HPA) axis, which is dysregulated—often hyperactive—in patients with major depressive symptoms [268,269]. It has been reported that ghrelin administration increases cortisol and growth hormone (GH), with only a weak trend toward mood improvement in male patients [270], and that ghrelin can alleviate anxiety after short-term stress by activating the HPA axis [271]. In line with these findings, rat studies using ghrelin O-acyltransferase (GOAT) inhibitors—thus reducing active (acyl-)ghrelin—showed dampened HPA activity, suggesting that active ghrelin modulates HPA tone and may affect depressive phenotypes [272]. In patients, ghrelin administration alters sleep architecture, increasing non-REM and reducing REM sleep [270,273]. In the same vein are recent results on the efficacy of the novel butyrylcholinesterase inhibitor (BChEI) in the Flinders Sensitive Line rat model of major depressive disorder. The application of BChEI improved behavioral-, cognitive-, and reward-related parameters of treated animals in a GHSR1a-dependent manner, likely by the increased protection of acyl-ghrelin from butyrylcholinesterase in serum [274].

Ghrelin also enhances dopaminergic signaling, and dysregulation of the dopamine system is linked to depressive symptoms [275]. Specifically, ghrelin activates ventral tegmental area (VTA) dopamine neurons, a mechanism also associated with appetite stimulation [276,277]. In mice, depressive-like behavior was reduced by ghrelin, an effect attributed to GHSR1a-dependent increases in dopamine levels and the promotion of dopaminergic neuronal responses and synapse formation [278,279]; these effects were at least partially reversed by administration of a ghrelin-receptor antagonist [280]. Preclinical evidence suggests that ghrelin inhibits serotonin synthesis via the nitric oxide (NO)-dependent suppression of tryptophan hydroxylase [281], a mechanism that may promote depressive phenotypes. Conversely, ghrelin enhances dopaminergic and orexin signaling [282,283], supporting reward processing, stress resilience, and emotional regulation. Recently, it was demonstrated that the ghrelin-independent constitutive activity of GHSR1a is required for the reversal (inhibition to excitation) of dopamine D2-receptor signaling in the spinal defecation center [284].

Stress elevates circulating ghrelin, modulating HPA axis activity and glucocorticoid release [285,286], although associations with cortisol in clinical studies remain inconsistent [287,288]. At the neuronal level, ghrelin stimulates hippocampal neurogenesis and synaptic plasticity through the PI3K/Akt–MAPK pathways, upregulating brain-derived neurotrophic factor (BDNF) [289,290]. Ligand-free (apo) GHSR1a can heteromerize with dopamine receptor D1 (DRD1), shaping reward-related plasticity [291,292].

Beyond its potential as a therapeutic target, ghrelin has been proposed as a biomarker to monitor antidepressant treatment response [293] and aid in the differential diagnosis between depression and bipolar disorder [294,295].

Disruption of ghrelin signaling is increasingly implicated in Alzheimer’s disease (AD). While total plasma ghrelin is often unchanged [296,297], some studies report elevated circulating levels in AD and prodromal AD, inversely correlating with cognitive performance [297,298]. This paradox may reflect reduced local ghrelin availability, as supported by decreased temporal lobe ghrelin mRNA [299]. Central to ghrelin signaling disruption in AD are alterations in ghrelin receptor (GHSR) expression and regulation. GHSR mRNA is reduced in leukocytes and the temporal lobes [298,299], yet hippocampal protein levels are increased, where Aβ directly binds and inhibits GHSR, impairing signaling [300]. GHSR1b, a splice variant that may suppress GHSR function, is also elevated in AD brains [299,301]. Additional modulation may involve liver-expressed antimicrobial peptide 2 (LEAP-2), which could exacerbate ghrelin resistance due to its association with obesity and metabolic syndrome, both recognized AD risk factors [302,303]. In recent years, ghrelin analogs were indeed demonstrated to have neuroprotective properties in vitro and in a AD triple-transgenic mouse model in vivo [304,305]. Ghrelin activation may also be altered through ghrelin O-acyltransferase (GOAT). Increased MBOAT4 (GOAT) mRNA has been observed in the leukocytes of AD patients [298], although its functional significance outside the gut remains unclear. Evidence for circulating “free” GOAT [31] raises additional questions about peripheral ghrelin activation in AD. Importantly, GOAT activity requires coenzyme A-activated fatty acids, especially medium-chain fatty acids (MCFAs), and dysregulated fatty acid metabolism in AD may further modulate ghrelin activation [306].

Recent data implicate the dysregulation of ghrelin signaling as a contributing factor in Parkinson’s Disease (PD). For example, a contemporary meta-analysis of studies from 2020 to 2024 (n ≈ 985 participants) shows markedly lower circulating levels of both total and acylated ghrelin in PD patients compared to healthy individuals. These differences are observed under fasting and postprandial states [307]. Acylated ghrelin exerts neuroprotective effects by promoting dopaminergic neuron survival through anti-apoptotic pathways (Bcl-2/Bax, caspase-3) [308,309]. It also reduces microglial-mediated neuroinflammation [90,309]. Ghrelin modulates mitochondrial function, oxidative stress, and systemic energy balance [310], potentially counteracting PD-associated metabolic disturbances [311]. The neuroprotective effects of ghrelin are dose- and context-dependent. Its conversion from acylated ghrelin to inactive des-acyl ghrelin (UAG) may reduce therapeutic efficacy. It has been recently suggested that nose-to-brain administration using gold nanoconjugates could overcome this limitation [312].

Ghrelin levels also correlate with non-motor PD features. Lower ghrelin concentrations are linked to gastrointestinal dysfunction, weight loss, and hyposmia, common symptoms of PD [313,314], while experimental ghrelin treatment improves GI motility and reduces dopaminergic neuron loss in animal models [309]. In addition, ghrelin receptor antagonism induces cataleptic behaviors and motor coordination dysfunction in mice. GHSR1a agonists have been proposed as potential therapeutic compounds to ameliorate both motor and non-motor symptoms in PD [315]. Together, these findings suggest that the decrease in ghrelin levels not only reflects disease progression, but may influence PD pathophysiology.

Overall, reduced ghrelin signaling is consistently associated with PD symptoms, and animal model data suggest that a dysfunctional ghrelin axis might be an indirect driver of neurodegeneration by increasing neuroinflammation and disturbing neuronal signaling. It must be kept in mind, however, that these mechanistic findings are mostly limited to rodent toxin-based models, which do not fully reflect the progression of human PD. Understanding how to restore or mimic ghrelin’s protective effects without disrupting metabolic homeostasis represents a promising avenue for future translational research, which may include optimized ligands and/or exploiting GHSR1a biased signaling.

Ghrelin signaling plays a central role in alcohol use disorder (AUD) [316]. In the brain, ghrelin affects reward-related regions, such as the amygdala [317], Edinger–Westphal nucleus [318], laterodorsal tegmental area, and lateral hypothalamus [319], modulating dopaminergic activity and reinforcing alcohol intake. Notably, chronic alcohol exposure upregulates GHSR expression within these structures [320], while ghrelin enhances alcohol-associated cues and neurochemical responses [321]. Preclinical studies show that ghrelin promotes alcohol intake, reward, and relapse primarily by stimulating the mesolimbic dopaminergic VTA–nucleus accumbens (VTA–NAc) circuit [322]. Conversely, the genetic deletion or pharmacological inhibition of GHSR1a (via antagonists or inverse agonists) consistently attenuate alcohol consumption, reduces motivational drive, and relapse-like behaviors across animal models [323,324]. Endogenous ghrelin dynamics appear context-dependent; acute alcohol administration reduces circulating ghrelin in male rats [325], whereas chronic exposure may lead to elevated levels [316]. Additional modulators of the ghrelin pathway, such as des-acyl ghrelin (DAG) and LEAP-2, may further influence addiction-related behaviors, although their roles remain less defined [326].

Beyond alcohol, ghrelin signaling broadly modulates the reinforcing properties of other addictive substances. In rodents, ghrelin enhances the locomotor activity, reward, and conditioned place preference (CPP) associated with psychostimulants (cocaine, amphetamines, nicotine), opioids (morphine, fentanyl, oxycodone), and cannabinoids [327,328,329,330,331,332]. Conversely, pharmacological inhibition of GHSR attenuates drug-induced dopaminergic transmission, CPP, self-administration, and reinstatement across these substance classes [324]. Complementary human studies indicate associations between GHSR gene polymorphisms and substance dependence, as well as positive correlations between circulating ghrelin levels and craving intensity [333].

Overall, ghrelin acts as a central regulator of appetite, energy balance, and neuroendocrine signaling within the nervous system. Ghrelin exhibits potent neuroprotective and anti-inflammatory effects, influences mood and stress regulation via the HPA axis and dopaminergic systems, and contributes to the pathophysiology of disorders such as depression, Alzheimer’s, Parkinson’s, and addiction. This poses ghrelin system as an attractive target for pharmacological interventions in the management of neurodegenerative disorders, neuroinflammation, and substance abuse.

## 6. GHSR1a Signaling Pathways

### 6.1. Ghrelin Is a Ligand for the Growth Hormone Secretagogue Receptor 1a (GHSR1a)

Ghrelin conveys its biological activity by binding to homo- and heterodimers of the growth hormone secretagogue receptor 1a (GHSR1a) (AG) and to yet-to-be-identified receptors (DAGs) [1,58]. GHSR1a exhibits widespread distribution across tissues and organs, including the lungs (especially in alveolar macrophages), kidneys, heart, liver, intestines, and adipose tissue [334,335]. Notable locations also include the ventromedial and arcuate nuclei of the hypothalamus, which govern feeding and body weight homeostasis [336]. Furthermore, a variety of immune cells, including monocytes, dendritic cells, B and T cells, and neutrophils, express GHSR1a on their surface [335,337].

Numerous studies indicate that an acyl group on Ser^3^ is essential for ghrelin’s biological activity through GHSR1a. The position of the octanoylated Ser is fundamental: moving the acyl group to Ser^2^ retains partial activity, while C8:0 at Ser^6^ or Ser^18^ leads to a reduction in activity. The fatty acid chain length also impacts receptor recognition, as maximal activity is retained by C10:0 Ser^3^, C12:0 Ser^3^, and C16:0 Ser^3^ ghrelin variants but is decreased in the case of the shorter C4:0 Ser^3^ or C2:0 Ser^3^ fatty acid modifications [40,338]. While substituting Ser^3^ with Trp^3^ preserves ghrelin activity, replacing it with aliphatic amino acids (Val, Leu, Ile) reduces it. The ester bond at Ser^3^ can be substituted with thioester or ether without affecting activity, highlighting the flexibility in chemical modifications [40]. The N-terminal positive charge and Phe^4^ are essential for activity and GHSR1a recognition. The minimal active fragment required for the activation of GHSR1a is the N-terminal pentapeptide, including C8:0 Ser^3^ [39]. Amidation of the C-terminus enhances, whereas N-acylation diminishes, activity [338,339]. Both acyl and des-acyl ghrelin exhibit a short α-helix conformation when bound to a lipid [340,341]. The minimal core sequence necessary for GHSR1a activation covers the N-terminal ^1^GSS_(Octanoyl)_FL^5^ sequence with indispensable octanoylated Ser^3^ in the middle (Figure 1).

Regardless of the identification of the short core ghrelin sequence required for receptor recognition, the interaction between AG and ghrelin-derived ligands is significantly more complicated than simple receptor stimulation and remains the subject of active research that aims to progress our understanding of multimodal ligand-based GHSR1a signal transduction.

### 6.2. GHSR1a and Biased Signaling

**Figure 3:**
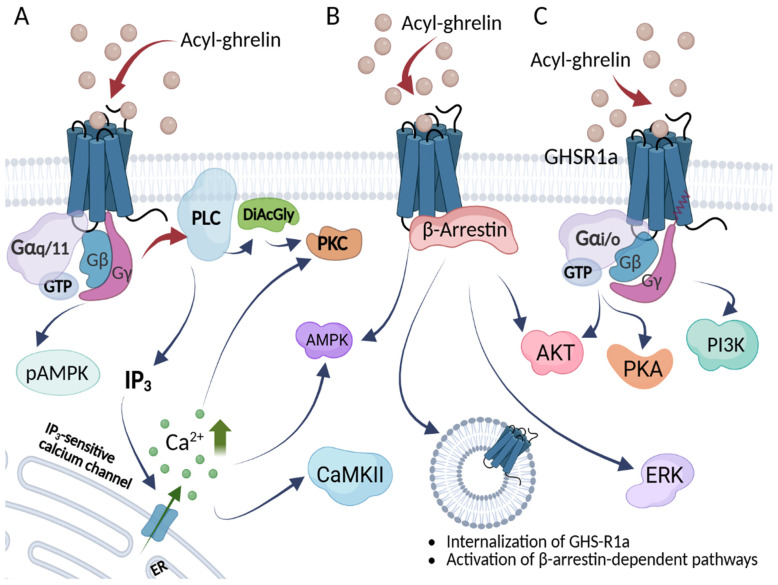
Signaling pathways associated with the ghrelin GHS-R1a receptor. Upon activation by ghrelin, GHSR1a engages Gαq/11 (**A**), Gαi/o (**C**), or β-arrestin (**B**) signaling. Each G-protein/β-arrestin complex is linked with a unique intracellular activation pathway and specific physiological effects. p: phosphorylated; AMPK: adeno-sine-monophosphate-activated protein kinase; DiAcGly: diacylglycerol; GTP: guanosine triphosphate; IP_3_: inositol 1,4,5-trisphosphate; PKC: Protein Kinase C; CaMKII: Ca^2+^/calmodulin-dependent protein kinase-IIa; PLC: phospholipase C; PI3K: phosphoinositide 3-kinase; PKA: protein kinase A; AKT: serine/threonine protein kinase; ERK: extracellular signal-regulated kinases. Created in BioRender. Kantyka, T. (2025) https://BioRender.com/atmjgad.

GHSR1a belongs to the A family of G-protein-coupled receptors (GPCRs), characterized by 7-transmembrane (7-TM) regions. Found in cell membranes, these receptors are selective for a wide range of ligands, from sophisticated proteins to small-molecule ligands like adrenaline. Classically, GHSR1a transduces the signal via GHSR1a-G_αq_-dependent pathway (Figure 3A), which leads to the increase in intracellular Ca^2+^, mediated by phospholipase C (PLC) activation and release of inositol 1,4,5-trisphosphate (IP3) and downstream activation of protein kinase C (PKC), Ca^2+^/calmodulin-dependent protein kinase-IIa (CamKII), and 5′ AMP-activated protein kinase (AMPK) [342,343,344]. In another G protein-related pathway (Figure 3C), GHSR1a–G_αi/o_ complex activates phosphoinositide 3-kinases (PI3K) to induce the activation of protein kinase A (PKA), PKCε, and serine/threonine protein kinase (AKT) [345,346]. Alternatively, G-protein-independent signaling is based on the signal relay via GSHR1a-β-arrestin complex (Figure 3B), leading to the increase in receptor internalization, accompanied by extracellular signal-regulated kinases (ERK1/2) and AKT activation [347,348]. It has been reported that the recruitment of G_αq/11_ is required for the regulation of food intake [349], while G_i2_ is necessary for the regulation of insulin release [350].

In recent years, significant progress has been made, and structures of GHSR1a with several ligands have been determined. Recognition of ghrelin and the antagonist Compound 21 involves the binding pocket, which bifurcates into two cavities separated by a salt bridge formed between TM3 E124^3.33^ and TM6 R283^6.55^, with cavity I created between the helices TM6 and TM7 of the receptor, which are dedicated to the binding of ghrelin N-termini and cavity II created between helices TM4 and TM5, recognizing the octanoylated side chain of Ser^3^ [351,352]. Further, the recent structure of GHSR1a with anamorelin, its small-molecule agonist, was currently investigated for cancer-related cachexia and anorexia treatment [353,354], confirming this mode of binding [355]. Notably, the study combined cryo-EM structural analysis and in-cell functional assays to elucidate the biased signaling of the GHSR1a. The repertoire of G-protein families recruited depends on the concentration and contact time of the ligands; different ligands (agonists/antagonists/reverse agonists) stabilize distinct receptor conformations, producing a characteristic G-protein/β-arrestin fingerprint [355,356]. These findings provide a structural explanation for GHSR1a-biased signaling and indicate that selected ligand combinations might be chosen for personalized treatment, even in the case of SNP variations in the receptor.

As progress in the clinical development of drugs targeting GHSR1a is relatively slow, new data on the pharmacology of GHSR1a ligands are needed to design new, more selective ligands with predictable biological activities. The pharmacology of GHSR1a is highly complex, involving G protein-dependent and -independent signaling pathways and high constitutive activity. The functional selectivity and signaling bias of many GHSR1a-specific ligands is still not well described. The activity of several peptides was investigated in respect to constitutive signaling, ligand-directed downstream GHSR1a signaling, functional selectivity, and signaling bias [357]. Biased ligand binding to GHSRs simultaneously stabilizes the receptor in a conformation that is able to selectively activate specific signaling pathways (Figure 3). Ghrelin can stabilize GHSRs in specific conformations that favor G-protein activation (G_αq_/G_αi/o_) or β-arrestin recruitment [358]. Interestingly, the activity of peptides (inverse agonist KwFwLL and agonist AwFwLL) depends on the key residues of transmembrane helices III and IV. Such mutations change the efficacy of KwFwLL from full inverse agonism in the WT receptor to partial agonism in the mutated receptor. In contrast, ghrelin stabilizes a different conformation that is able to recruit G_αq_, G_αi/o_, and β-arrestin, but not G_αs_. These findings are in line with the finding that the GHSR conformation induced by ghrelin in the presence of β-arrestin differs from ghrelin-induced conformation in the presence of G_αq_ [359]. Indeed, GHSR exhibits distinct conformations when activated by different ligands. There is a growing collection of agonists and antagonists for GHSR, demonstrating a spectrum of activities. Ramirez et al. examined a group of biologically active substances, including the following: ghrelin, Ibutamoren (MK-0677), L692,585, and [D-Lys3]-growth hormone–releasing peptide-6 (DLys3-GHRP-6), JMV2959, and [D-Arg^1^,D-Phe^5^,D-Trp^7,9^,Leu^11^]-substance P (SP-analog) [360]. DLys3-GHRP-6 behaved as a partial antagonist, with a strong bias toward GHSR1a–β-arrestin signaling, whereas JMV2959 acted as a full unbiased GHSR1a antagonist. Moreover, the SP-analog at high concentrations behaved as an inverse agonist increasing G-protein-dependent signaling, whereas at low concentrations, the SP-analog attenuated β-arrestin-dependent signaling (Table 1).

Liver-expressed antimicrobial peptide 2 (LEAP-2) was first described as an antimicrobial peptide expressed in the liver [361]. Subsequent investigation revealed that LEAP-2 is an endogenous antagonist of GHSR1a blocking the action of ghrelin by competing at the ghrelin-binding site [362]. LEAP-2 increases whereas ghrelin decreases in obesity, and LEAP-2 is implicated in obesity-related disorders, including polycystic ovary syndrome and non-alcoholic fatty liver disease [363]. Recent studies have revealed that ghrelin attenuates obesity-induced myocardial injury through various signaling axes [183,184]. It has been shown that knockdown of LEAP-2 relieved hyperlipidemia, inflammation, and myocardial injury in obese mice by polarizing macrophages toward the M2 phenotype [185]. LEAP-2 is being investigated as a potential therapeutic target for obesity and related metabolic diseases, as it can impact food intake and body weight [364]. Recent studies have focused on developing LEAP-2 analogs with potential clinical application [365]. For a summary of the modes of action and strategies for the modulation of ghrelin receptors in pharmacological contexts, please refer to Table 2. Progress in the clinical trials of ghrelin-receptor ligands has been recently updated in a review by Bukhari [366].

Despite the complicated landscape of ghrelin signaling pathways, our understanding of the receptor structure and biased signaling is progressing. As of now, only anamorelin, a small-molecule synthetic agonist of GHSR1a, has found limited clinical use in cachexia treatment of cancer patients.

**Table 1:** Selected ligands of the human growth hormone secretagogue receptor (GHSR1a).

Ligand	Type	Activity	Signaling Pathways				Selected Refs.
			Ca^2+^ Mobilization	β-Arrestin	GHSR1a Intern.	ERK Phosph.	
Ghrelin (human, acylated)	Endogenous peptide	Full agonist (canonical)	+	+	+	+	[1,360]
Des-acyl ghrelin (DAG)	Endogenous peptide (des-acyl)	Weak/low-potency agonist in vitro; often functionally GHSR1a-independent in vivo					[39]
Mini-ghrelins (1–15, 1–14, 1–11)	Endogenous peptide fragments	Competitive antagonists					[38,39]
LEAP-2	Endogenous peptide/protein	Competitive antagonist					[362]
Anamorelin	Small-molecule	Potent agonist					[355,367]
Ibutamoren (MK-677)	Small-molecule	Potent, selective, orally active agonist	+	+	+	+	[360,368,369]
			+	+	+	+	
L-692,585	Small-molecule	Agonist					[360,370,371]
JMV2959	Small-molecule	Unbiased antagonist; bias-inverse agonist	+/−		Basal −	0	[360,372]
Compound 21 (C21)	Small-molecule	Neutral antagonist					[351]
PF-5190457	Small-molecule	Orally active inverse agonist					[333,373]
					Basal −	0	
[D-Lys^3^]-GHRP-6	Peptide analog	Preferentially β-arrestin pathway blocker; bias-inverse agonist					[360,374,375]
Substance P analog (D-Arg^1^,D-Phe^5^,D-Trp^7,9^,Leu^11^-SP)	Peptide analog	Inverse agonist at higher concentrations; attenuates β-arrestin at low concentrations			Basal −	Basal −	[163,360]
KwFwLL	Peptidomimetic	Inverse agonist					[376]
AwFwLL	Peptidomimetic	Agonist					[376]

GHSR1a intern.: Internalization of the receptor; ERK Phosph.: Extracellular signal-regulated kinase phosphorylation. Activity symbols: Basal −: Reduction in the constitutive (basal) receptor activity; +: increase in signal; 0: no effect; +/− weak increase in signal.

**Table 2:** Translational potential of ghrelin-system modulating compounds.

Target/Strategy	Modality	Representative Ligand(s)	Intended/Observed Effect(s)	Implementation	Reference
GHSR1a activation	Small-molecule agonists	Anamorelin; Ibutamoren (MK-677); L-692,585	Appetite/weight gain; GH axis activation; pro-anabolic effects	Preclinical + clinical signals (anamorelin); preclinical/pharmacology for MK-677, L-692,585	[355,360,367,368,369,370,371]
GHSR1a neutral antagonism	Small-molecule antagonist	Compound 21 (*C21*)	Blocks receptor without inverse signaling	Preclinical/pharmacology	[351]
GHSR1a inverse agonism	Small-molecule and peptidomimetic inverse agonists	PF-5190457 (oral clinical candidate); KwFwLL (peptidomimetic)	Suppresses high constitutive activity; pathway-selective effects	Preclinical + early clinical (PF-5190457)	[163,333,373]
β-arrestin–pathway blockade/biased modulation	Peptide analog (biased)	[D-Lys^3^]-GHRP-6	Preferentially blocks β-arrestin signaling; bias-inverse actions	Preclinical/pharmacology	[360,374,375]
Concentration-dependent inverse agonism/signaling reweighting	Peptide analog	Substance P analog (D-Arg^1^,D-Phe^5^,D-Trp^7,9^,Leu^11^-SP)	Inverse agonist at higher doses; attenuates β-arrestin at lower doses	Preclinical/pharmacology	[163,360]
Endogenous antagonism of GHSR1a	LEAP-2 (native) and LEAP-2 analogs	LEAP-2; truncated palmitoylated LEAP-2 analog, LA-LEAP2 analog	Antagonizes ghrelin; reduces food intake/weight in models; improves obesity-related injury via immune effects	Preclinical + translational rationale; analogs preclinical	[185,362,363,364,365,377]
Competitive peptide antagonism	Endogenous fragments (“mini-ghrelins”)	Ghrelin(1–15), (1–14), (1–11)	Competitive antagonists at GHSR1a; block orexigenic effects in vivo	Preclinical	[38,39]
Enzymatic pathway modulation	Prospective GOAT inhibitors	BI 1356225	Reduce acyl-ghrelin generation; shift AG/DAG balance	Conceptual/prospective in early clinical development	[42,65,378]
Pathway-selective (“biased”) ligand design	Prospective ligand discovery leveraging receptor structures	concept	Tailor G-protein vs. β-arrestin signaling; reduce off-target effects	Conceptual/mechanistic rationale; structural and functional bases	[355,356,357,360]

Ghrelin represents a highly conserved signaling molecule that coordinates metabolic, fertility, cardiovascular, and immune regulation across multiple vertebrate species. Its octanoylation by the acyltransferase GOAT enables GHSR1a receptor binding, which in turn mediates a broad spectrum of biological effects extending far beyond its initial identification as an orexigenic hormone. The presence of both the acylated and des-acyl ghrelin forms affects multiple aspects of physiological regulation and homeostasis maintenance on a not-yet-understood level. Although des-acyl ghrelin (DAG) remains a far less potent stimulant of GHSR1a, the local, possibly extracellular, re-acylation or GHSR1a receptor-independent DAG ability to activate intracellular survival and metabolic signaling call for further exploration.

Ghrelin serves as a coordinator of energy homeostasis, linking nutrient sensing with mitochondrial health, immune modulation, reproduction regulation, and cardiovascular fitness. It promotes oxidative metabolism, limits apoptosis, and regulates macrophage polarization, maintaining tissue integrity and homeostasis under stress. In the cardiovascular system, ghrelin safeguards endothelial function and myocardial performance, while it acts as a metabolic gatekeeper in adolescence and reproduction. In the nervous system, ghrelin exerts neuroprotective effects, affecting reward, mood, and cognitive processes through hypothalamic and dopaminergic circuits and by neuroinflammatory mediation.

At the molecular level, the discovery of biased signaling of the GHSR1a receptor and the recent structural description of ligand–GHSR1a complexes have unveiled novel approaches for rational ligand design. Distinct receptor conformations selectively engage the G-protein or β-arrestin pathways, offering a unique opportunity for the development of pathway- or tissue-selective therapeutics. Pharmacological tools, including inverse and bias-selective agonists, currently developed GOAT inhibitors, and small-molecule agonists, already translate into an unprecedented arsenal of strategies that target the ghrelin axis, and call for further development.

Regardless of this recent progress, multiple questions still remain unanswered. Identification of the precise physiological role of DAG, the mechanisms of GOAT regulation and extracellular activity, and the impact of post-translational ghrelin modifications on receptor selectivity require further investigation. Similarly, the effects of ghrelin on the immune system under normal, acute, and chronic inflammatory conditions requires further work to fully elucidate the effects of ghrelin on the inflammatory milieu, possibly adding to our understanding of persisting inflammatory states.

Future research will focus on delineating tissue-specific ghrelin signaling networks, structural determinants of biased ligand signal transduction, and the long-term clinical safety of ghrelin-targeted drugs that target different physiological aspects with individualized, optimized strategies. The integration of structural biology, pharmacology, and translational studies will be essential to harness the full therapeutic potential of this multifaceted hormone, not only for the obesity treatment in the post-GLP-1 receptor agonist era, but also in the management of inflammatory diseases, neuropsychiatric disorders, and addiction-related therapy.

## Data Availability

No new data were created or analyzed in this study. Data sharing is not applicable to this article.

## Abbreviations

The following abbreviations are used in this manuscript:

5-HD	5-hydroxydecanoate
ACC	acetyl-CoA carboxylase
AD	Alzheimer’s disease
AEBSF	4-(2-aminoethyl)benzenesulfonyl fluoride hydrochloride
AG	acyl ghrelin
AgRP	agouti-related peptide
AKT	Protein kinase B; serine/threonine protein kinase
AMPK	5′ AMP-activated protein kinase
APC	activated protein
APT1	Acyl-Protein Thioesterase 1/Lysophospholipase
ARC	arcuate nucleus
Arg-1	arginase-1
AUD	alcohol use disorder
BBB	blood–brain barrier
BChEI	butyrylcholinesterase inhibitor
BDNF	brain-derived neurotrophic factor
CamKII	Ca^2+^/calmodulin-dependent protein kinase-IIa
CART	cocaine- and amphetamine-regulated transcript
CKD	chronic kidney disease
CNS	central nervous system
CoA	Coenzyme A
CPP	conditioned place preference
CPT1	carnitine palmitoyltransferase-1
CREB	cAMP response element-binding protein
CRTC1/TORC1	CREB-regulated transcription coactivator 1/Transducer Of Regulated CREB activity 1
Cx43	connexin 43
DAG	des-acyl ghrelin
DiAcGly	Diacylglycerol
DLys3-GHRP-6; D-Lys3	[D-Lys3]-growth hormone–releasing peptide-6
DMN	dorsomedial nucleus
DRD1	dopamine receptor D1
EDTA	Ethylenediaminetetraacetic acid
ER	endoplasmic reticulum
ERK1/2	extracellular signal-regulated kinase ½
FAO	fatty acid oxidation
FBS	fetal bovine serum
FNDC5	Fibronectin type III domain-containing protein 5
FSH	follicle-stimulating hormone
GABA	gamma-aminobutyric acid
GCF	gingival crevicular fluid
GH	growth hormone
GHSR1a	growth hormone secretagogue receptor 1a
GHSR1b	growth hormone secretagogue receptor 1b
GLP-1	Glucagon-like peptide-1
GLUT4	Glucose transporter type 4
GLUT5	Glucose transporter type 5
GnRH	gonadotropin-releasing hormone
GOAT	ghrelin O-acyltransferase
GPCR	G protein-coupled receptor
HMGB1	High Mobility Group Box 1
HPA	hypothalamus–pituitary–adrenal
HPG	hypothalamic-pituitary-gonadal axis
IGF-1	insulin-like growth factor-1
IL-1β	Interleukin-1 beta
iNOS	inducible nitric oxide synthase
IP3	inositol 1,4,5-trisphosphate
iPSC	Induced pluripotent stem cells
IRI	ischemia–reperfusion injury
IRS1	Insulin receptor substrate 1
IRS2	Insulin receptor substrate 2
LC3-I/LC3-II	microtubule-associated proteins 1A/1B light chain 3 I/II
LEAP-2	Liver-expressed antimicrobial peptide 2
LH	luteinizing hormone
LHA	lateral hypothalamic area
LPS	Lipopolysaccharide
MAFP	methoxy arachidonyl fluorophosphonate
MBOAT	membrane-bound-O-acyltransferase
MCP1	monocyte chemoattractant protein 1
MCP-1	monocyte chemoattractant protein-1
MDA	malondialdehyde
Mgl-1	macrophage galactose-type lectin-1
mitoKATP	mitochondrial ATP-sensitive potassium channels
MSCs	mesenchymal stem cells
mtTFA	mitochondrial transcription factor A
NAc	nucleus accumbens
NAFLD	non-alcoholic fatty liver disease
NASH	non-alcoholic steatohepatitis
NF-κB	Nuclear factor kappa-light-chain-enhancer of activated B cells
NMN	nicotinamide mononucleotide
NO	nitric oxide
NPY	neuropeptide Y
P62	ubiquitin-binding protein P62
PA	palmitic acid
PC	prostate cancer
PC1/3	prohormone convertase 1/3
PCSK1	prohormone convertase 1/3
PD	Parkinson’s Disease
PGC-1α	Peroxisome proliferator-activated receptor gamma coactivator 1-alpha
PGC-1β	Peroxisome proliferator-activated receptor gamma coactivator 1-beta
PGE2	Prostaglandin E2
PGF2α	Prostaglandin F2α
PI3K	Phosphoinositide 3-kinase
PKA	protein kinase A
PKC	protein kinase C
PKCε	protein kinase Cε
PLC	phospholipase C
PMSF	phenylmethylsulfonyl fluoride
POMC	proopiomelanocortin
PPARα	peroxisome proliferator-activated receptor alpha
PPARγ	peroxisome proliferator-activated receptor gamma
PVN	paraventricular nucleus
RIA	radioimmunoassay
ROS	reactive oxygen species
scRNA-seq	Single-cell RNA sequencing
SNP	single-nucleotide polymorphism
SP-analog	[D-Arg^1^,D-Phe^5^,D-Trp^7, 9^,Leu^11^]-substance P
TBI	traumatic brain injury
TLR	Toll-like receptor
TM	transmembrane helix
TNF-α	Tumor necrosis factor alpha
UCP2	uncoupling protein-2
UCP3	Mitochondrial uncoupling protein 3
VEGF	Vascular endothelial growth factor
VMN	ventromedial nucleus
VTA	ventral tegmental area
